# The Efficacy of Fenofibrate in Addition to Atorvastatin in Patients of Type II Diabetes Mellitus

**DOI:** 10.7759/cureus.22852

**Published:** 2022-03-04

**Authors:** Muhammad Qasim, Abroo Bahadur, Shafi Ullah Khan, Adnan Rahman, Aqil Noor, Maaz Zafar, Kiran Abbas

**Affiliations:** 1 Endocrinology and Diabetes, Hayatabad Medical Complex (HMC), Peshawar, PAK; 2 Department of Gynecology and Obstetrics, Mian Rashid Hussain Shaheed Memorial Hospital Pabbi, Nowshera, PAK; 3 Department of Medicine, District Headquarter Hospital Kohat Development Authority (KDA), Kohat, PAK; 4 Department of Dentistry and Periodontology, Peshawar Dental College, Peshawar, PAK; 5 Department of Medicine, Jinnah Postgraduate Medical Centre, Karachi, PAK

**Keywords:** saroglitazar, fenofibrate, efficacy, diabetes mellitus, atorvastatin

## Abstract

Background

The objective was to study the efficacy of atorvastatin in combination with fenofibrate as compared to atorvastatin in combination with saroglitazar in patients of diabetes mellitus type II with dyslipidemia.

Methodology

A quasi-experimental study was done at the Diabetes and Endocrinology Ward, Hayatabad Medical Complex Peshawar, between January 2021 to June 2021. All patients aged 25 years and above with newly diagnosed diabetes mellitus (less than six months ago) with dyslipidemia, i.e., deranged lipid range, were eligible to participate. Patients with secondary hypertension, pregnancy, or any pulmonary disease were excluded from the study. Patients already taking anti-glycemic drugs were also ineligible to participate. Patients were divided into two groups. Group I patients received Atorvastatin 10mg plus Fenofibrate 145 mg, while Group II received the combination of the tab. Atorvastatin 10mg in addition to Saroglitazar 4g. Lipid profiles were studied at baseline and 24-month follow-up. All data were documented in a preformed proforma.

Results

A total of 80 patients were enrolled in the study, with 40 patients in each group. In Group I (atorvastatin + fenofibrate), the mean cholesterol at 24-week follow-up was 254.51 ± 47.41 as compared to 230.45 ± 47.21 in Group II (p<0.0001). Similarly, total triglycerides, low-density lipoprotein (LDL), and very-low-density lipoprotein (VLDL) were significantly higher in Group I patients by 24-week follow-up as compared to Group II. The mean HDL levels in Group I changed from 40.21 ± 3.54 at baseline to 46.28 ± 6.25 at follow-up, while in Group II, the mean HDL levels altered from 39.54 ± 4.52 to 52.34 ± 7.54 (p<0.0001).

Conclusion

Overall, both groups showed significant improvements in lipid profiles; however, when atorvastatin in addition to fenofibrate was compared with saroglitazar, it was found that the latter combination was more effective in improving the overall patient outcome.

## Introduction

Globally, diabetes mellitus is among the most prevalent chronic illnesses, and its burden is in an increasing trend. It is one of the leading causes of mortality. In the next five to seven years, the proportion of individuals with diabetes may increase by 69% in low-middle income nations and 20% in industrialized regions [1-3].

Generally, diabetes and its associated dyslipidemia have often been addressed with a combination of conventional anti-diabetic agents (ADAs) and hypolipidemic therapies [4-5]. The benefits of statins are only limited to up to 20% to 30% of patients in cases of dyslipidemia. Moreover, niacin-induced myotoxicity and lack of efficacy of fibrates create a therapeutic limitation and therefore remain unsuccessful agents [6,7].

To overcome the treatment gaps, the molecules of Peroxisome Proliferator-Activated Receptors (PPAR)-α/ γ agonists. Studies suggest that these compounds are highly potential to address both hyperglycemia and dyslipidemia in diabetic patients [8]. The use of fenofibrate in combination with a statin has been recommended as a potential therapeutic option for better lipid profiles and minimizing cardiovascular risk.

Furthermore, combining statins and fibrates is more effective in limiting atherogenic dyslipidemia as compared to monotherapy [9]. Fenofibrate induces an elevation in lipase activity and a reduction in cholesterol ester transport protein function via activating peroxisome proliferator-activated receptors [10]. The outcome is a decline in triglycerides (TG) levels, a change in low-density lipoprotein (LDL) particle size, and an increase in high-density lipoprotein (HDL) cholesterol.

The considerable decrease in TG and rise in HDL observed in this investigation with the combination of atorvastatin and fenofibrate indicates a significant increase in LDL particle size.

Hence there is a need to thoroughly know the implications of combining statins and fenofibrate on the characteristics of dyslipidemia and appropriately estimate cholesterol control in our population. The focus of this research was to determine how efficiently statins work in lowering dyslipidemia in a Pakistani population with type II diabetes. The prime goal of the study was to evaluate the effectiveness of atorvastatin in addition to fenofibrate to atorvastatin in combination with saroglitazar in individuals with type II diabetes and dyslipidemia.

## Materials and methods

A quasi-experimental study was undertaken at the Diabetes and Endocrinology Ward, Hayatabad Medical Complex Peshawar, between January 2021 to June 2021. A non-probability convenience technique was employed to recruit individuals into the study. Ethical clearance was obtained before the data acquisition.

The sample size of 80 (40 in each group) was calculated using select statistics software by keeping the mean change in total cholesterol levels after treatment to be 173 ± 5 mg/dl in patients on Atorvastatin versus 171 ± 4 mg/dl in patients who were administered Atorvastatin plus Fenofibrate [9]. 

All individuals between the ages of 25 years and above, with newly diagnosed diabetes mellitus (less than six months ago) with dyslipidemia, i.e., deranged lipid range, according to the National Cholesterol Education Program, were eligible to participate. Patients with secondary hypertension, pregnancy, or any pulmonary disease were excluded from the study. Patients already taking anti-glycemic drugs were also ineligible to participate. 

After fulfillment of inclusion criteria and informed written consent was obtained from patients after that study was started. The participants were categorized into two classes. Group I patients received tab. Atorvastatin 10mg + Tab. Fenofibrate 145mg while Group II received the combination of the tab. Atorvastatin 10mg in addition to Saroglitazar 4g. Lipid profiles were studied at baseline and 24-month follow-up. For the documentation, a preformed questionnaire was utilized. 

The analysis was performed on the IBM Corp. Released 2017. IBM SPSS Statistics for Windows, Version 25.0. Armonk, NY: IBM Corp. For scalar parameters like age, the mean and standard deviation was determined, and for categorical variables, frequency and percentages were used. The efficacy of the therapy was defined by a significant change observed in clinical parameters between the classes. A student t-test was applied to check the significance. For statistical significance, a p-value of below 0.05 was decided. 

## Results

A total of 80 individuals were recruited in the study and were equally divided into groups. The mean age of patients was 42.5 (13.21) years. There was a female predominance (Table 1). 

**Table 1:** Participant Characteristics

Parameters	Groups	
	Group I	Group II
Age in years		
30 to 40	11 (27.50%)	12 (30.00%)
40 to 50	16 (40.00%)	17 (42.50%)
50 to 60	13 (32.50%)	11 (27.50%)
Gender		
Men	17 (42.45%)	17 (42.50%)
Women	23 (57.55%)	23 (57.50%)

In Group I (atorvastatin + fenofibrate), the mean cholesterol at 24-week follow-up was 254.51 ± 47.41 as compared to 230.45 ± 47.21 in Group II (p<0.0001). Similarly, total triglycerides, LDL, and VLDL were significantly higher in Group I patients by 24-week follow-up as compared to Group II. The mean HDL levels in Group I changed from 40.21 ± 3.54 at baseline to 46.28 ± 6.25 at follow-up, while in Group II, the mean HDL levels altered from 39.54 ± 4.52 to 52.34 ± 7.54 (p<0.0001) (Table 2). 

**Table 2:** Comparison of Parameters in Both Groups at Baseline and at 24 weeks of treatment

Parameters	Group		p-value
	Group I (Fenofibrate)	Group II (Saroglitazar)	
Total Cholesterol			
At baseline	301.64± 58.41	308.44 ± 50.21	0.356
At 24-week	254.51 ± 47.41	230.45 ± 47.21	<0.0001
Total Triglycerides			
At baseline	244.21 ± 54.47	245.75 ± 70.32	0.878
At 24-week	212.56 ± 80.54	173.21 ± 56.58	0.0001
HDL			
At baseline	40.21 ± 3.54	39.54 ± 4.52	0.252
At 24-week	46.28 ± 6.25	52.34 ± 7.54	<0.001
LDL			
At baseline	215.54 ± 49.41	217.55 ± 50.56	0.465
At 24-week	174.44 ± 51.45	141.54 ± 50.58	<0.0001
VLDL			
At baseline	50.14 ± 9.54	51.54 ± 12.41	0.544
At 24-week	40.12 ± 17.50	33.74 ± 12.14	<0.0001

Overall, both groups showed significant improvements in lipid profiles; however, when Group I was compared with Group II, it was found that significantly greater improvements were observed in Group II. 

## Discussion

Dyslipidemia resulting in severe cardiovascular diseases is a common complication in diabetes mellitus. Lipid-lowering medical therapy is an effective intervention for achieving a target lipid profile, with the first line recommended agent being statins [9]. The present study evaluated the effectiveness of statins in combination with fenofibrate and saroglitazar in improving lipid levels among patients with diabetes mellitus. The results revealed that though improvements in lipid levels were observed in both groups of drugs, combination therapy using statin and saroglitazar showed much more significant improvements in lipid profiles.

The current study revealed a more significant reduction in LDL levels in patients treated with saroglitazar compared with fenofibrate. Similar to this study, a randomized control trial was conducted by Parmar et al., which assessed the efficacy of saroglitazar and fenofibrate. The study found that both drugs were effective in achieving target lipid levels [10]. However, saroglitazar was more effective than fenofibrate in controlling lipid levels. This finding was consistent with our current study, which reported similar results. However, our findings were inconsistent with the study performed by Davidson et al., in which a more significant reduction in LDL was seen in patients treated with fenofibrate and statins instead of saroglitazar [11].

An identical study was also conducted by Patel et al., which concluded that fenofibrate was more effective in reducing the levels of VLDL as compared to saroglitazar when used in combination with statins. However, our findings were contradictory to this study, and it was revealed that reduction in levels of VLDL in patients belonging to Group II was statistically more significant as compared with those in Group I [12].

The effectiveness of fenofibrate was studied in The Action to Control Cardiovascular Risk in Diabetes (ACCORD) Lipid randomized control trial involving 5518 patients suffering from diabetes mellitus. The results revealed no significant difference between patients treated with fenofibrate and statin and statin alone [13]. This implied that no additional benefit was observed in patients treated with combination therapy.

However, another randomized controlled trial comprising 9795 patients revealed contrary findings in the Fenofibrate Intervention and Endpoint Lowering in Diabetes (FIELD) study. It was found that a combination of statins with fenofibrate offered a 40% reduction in relative risk of complication in diabetes mellitus secondary to dyslipidemia. The study concluded that fenofibrate was highly protective against cardiovascular events [14].

Kim et al. studied HDL and LDL cholesterol levels in patients and evaluated the changes in baseline readings in patients treated with statin and fenofibrate and statin alone. The study found that combined therapy was associated with a more significant reduction in triglyceride levels, thereby supporting the efficacy of fenofibrate in the treatment of dyslipidemia [15]. Interestingly, quite coinciding findings were revealed by Zhao et al., who concluded that following treatment with fenofibrate, there was a significant reduction in triglyceride levels and a marked increase in total HDL [16]. The Study for Atrial Fibrillation Reduction (SAFARI) trial also observed these findings, which saw a 23.6% reduction in triglyceride levels and an 8.8% increase in HDL following combined use of statin and fenofibrate [17,18].

The current study was limited due to its small sample size. Research on a larger scale must be conducted to understand the efficacy of treatment better. More research focusing on lipid-lowering therapy is warranted.

## Conclusions

Diabetes and hyperlipidemia are linked with significant complications, thereby emphasizing lipid-modifying agents as potential medical options. Our study concluded that statins in combination with fenofibrate and saroglitazar are both effective combination therapies against the progression of diabetes mellitus. Overall, both groups showed significant improvements in lipid profiles; however, when atorvastatin in addition to fenofibrate was compared with atorvastatin plus saroglitazar, it was found that the latter combination was more effective. 
